# *ANKHD1* promotes pathogenic proliferation in Autosomal Dominant Polycystic Kidney Disease via the Cyclin D1/CDK4 pathway

**DOI:** 10.1186/s12967-025-06359-9

**Published:** 2025-06-02

**Authors:** Maria-Eirini Terzenidou, Foteini Patera, Fiona M Macleod, Albert C M Ong, Maria Fragiadaki

**Affiliations:** 1 William Harvey Research Institute, Queen Mary University of London, Charterhouse Square, London, UK; 2 Division of Physiology, Pharmacology and Neuroscience, School of Life Sciences, University of Nottingham, Nottingham, UK; 3 Centre for Cancer Sciences, School of Medicine, Biodiscovery Institute, University of Nottingham, Nottingham, UK; 4 Academic Nephrology Unit, Division of Clinical Medicine, School of Medicine and Population Health, University of Sheffield, Sheffield, UK

## Abstract

**Background:**

Autosomal Dominant Polycystic Kidney Disease (ADPKD) is the most common genetic cause of renal failure. Uncontrolled proliferation drives ADPKD, which manifests with cystic kidney enlargement. Yet, the mechanisms by which renal epithelial cells lose cell cycle control are largely unknown. To investigate this, we examined the expression and function of the Ankyrin Repeat and single KH Domain 1 (ANKHD1), which positively regulates proliferation in cancer, yet its role in ADPKD is unexplored.

**Results:**

We report elevated proliferation (Ki67 and Cyclin D1) in three independent mouse models of ADPKD, the *Pkd1*^*nl/nl*^, the *Pax8-cre; Pkd1*^*del/del*^ and the *KSP-cre; Pkd1*^*del/del*^. We find that ANKHD1 protein localises in cyst lining cells of both aquaporin-1 and 2 (AQP1-AQP2) positive cysts. ANKHD1 knockdown in human cells or knockout in mouse tissues resulted in reduced proliferation, slower cystic growth in vitro and smaller kidneys in vivo; ultimately leading to improved renal function. Mechanistically, ANKHD1 binds to CDK4 and positively controls the Cyclin D1/CDK4 pathway. ANKHD1-mediated enhancement of Cyclin D1/CDK4 activity leads to increased retinoblastoma phosphorylation and proliferation, a mechanism that is p19-dependent but p21 independent.

**Conclusions:**

We report a functional role for ANKHD1 in driving pathogenic proliferation in ADPKD via the Cyclin D1/CDK4 axis.

## Background

Autosomal Dominant Polycystic Kidney Disease (ADPKD) is an adult-onset condition affecting multiple organs including the kidneys, where multiple fluid-filled cysts are formed [1–3]. ADPKD is the most common genetic cause of renal failure and arises mainly due to mutations in *PKD1* gene, which encodes polycystin-1, a multidomain integral membrane protein [4]. A minority of patients present with mutations in a related gene, *PKD2* or polycystin-2 [5], a Ca^2+^-permeable ion channel protein. The polycystin1/2 proteins form multimeric complexes and modulate several signalling pathways affecting proliferation, apoptosis, fluid transport, cell adhesion and differentiation [6, 7]. ADPKD has been proposed to develop when the polycystin levels drop below a critical threshold [8–11]. This loss of heterozygosity/dosage effect often takes place in adulthood and coincides with the onset of symptoms, with both the timing and the dose of *PKD1* being important in controlling the onset and severity of the disease [8, 12]. One potential mechanism for lowering the dose of polycystins is explained by the two-hit hypothesis of cyst formation; whereby *PKD1* or *PKD2* mutations in one allele are inherited in the germline and a second-hit event takes place later in life, often a somatic mutation in the remaining allele, that triggers the onset of symptoms [13–15].

The pathogenesis of ADPKD is characterised by the progressive development of renal cysts leading to kidney hypertrophy, which in turn leads to kidney failure. While the mechanisms underlying ADPKD are multifaceted, dysregulated cell proliferation plays a crucial role in both the initiation and progression of the disease [16]. Loss of function mutations in *PKD1* or *PKD2* gene are accompanied by cellular de-differentiation [8, 9, 17] and aberrant proliferation in renal tubular epithelial cells [18–21]. This uncontrolled cell division, coupled with altered apoptosis, contributes to cyst formation and expansion. Recent studies have further refined our understanding by showing that control of the proliferative axis can reduce cyst size and improve renal function [22, 23], suggesting that proliferation is at the heart of ADPKD. This is emphasised by next-generation sequencing studies that report proliferation as one of the most upregulated pathways in ADPKD [16, 24]. The impact of PKD mutations leading to uncontrolled cell proliferation extends beyond the kidneys with extrarenal manifestations, including enlarge polycystic liver disease and intracranial aneurysms [25]. Hence of understanding the mechanisms that underlie proliferation control are likely to provide benefit across multiple organ systems.

Many signalling pathways have been suggested to regulate cyst growth at least in part by altering cell proliferation. The mTOR pathway, has been shown to be regulated by polycystin-1, when inhibited with rapamycin, leads to reduced renal cystogenesis in PKD models [26]. The MAPK/ERK pathway is activated in PKD leading to increased glycolysis and altered metabolism towards the Warburg effect, hence maintaining abnormal cell proliferation, contributing to cyst growth [27]. Also, levels of cAMP are increased in numerous animal models of PKD [28], leading to protein kinase A (PKA) and ERK pathway activation, resulting in increased proliferation [29]. Furthermore, the Hippo-YAP/TAZ signalling pathway, which is upregulated in ADPKD [30], was recently shown to be linked to excessive proliferation in ADPKD [31], but the mechanism leading to altered Hippo activity was not addressed. Cyclin-dependent kinase 1 (CDK1), a positive regulator of proliferation, is upregulated in ADPKD, while its knockdown improves kidney function [24]. Lastly, JAK2/STAT1 was shown to interact with PKD1/PKD2 to become activated by phosphorylation in renal epithelial cells and promote quiescence [32]. Taken together, the mTOR, PKA/MAPK/ERK, cAMP, Hippo, JAK/STAT and CDK1 pathways have all been implicated as drivers of proliferation in ADPKD, yet how they are controlled is currently unknown.

RNA-binding proteins (RBPs) play pivotal roles in regulating gene expression and influencing cellular processes [33], including those implicated in ADPKD pathogenesis, such as cyst formation [34]. Ankyrin repeat and Single KH Domain 1 (ANKHD1) is an RBP that binds various RNA species [35, 36] and has been identified as a regulator of signalling pathways relevant to ADPKD, including the Hippo [37], JAK/STAT [38] and CDK pathways [39]. These pathways are known to influence cell proliferation and cyst growth in ADPKD. However, the specific role of ANKHD1 in regulating proliferation in polycystic kidneys remains unknown. By targeting ANKHD1 we have the opportunity to target multiple pathways involved in ADPKD, and therefore maximise the therapeutic potential. We hypothesise that ANKHD1 plays a role in ADPKD by controlling the way epithelial cells with *PKD1* mutations proliferate. Here we present the first mechanistic study identifying ANKHD1 as a critical regulator of proliferation in ADPKD.

## Methods and materials

### In vivo studies

All animal experiments were performed under the authority of a United Kingdom Home Office license and in accordance with the UK Animal Scientific Procedures Act (1986). The heterozygous *Pkd1*^*nl/nl*^ murine model and *iKSPCreER*^*T2*^*/Pkd1*^*fl/fl*^ tissue-specific KO mouse model were obtained from the Dorien Peters laboratory [12] and were maintained with heterozygote breeding. Mice that were either wild-type or nl/nl for the Pkd1 locus were used for experiments. *iKSPCreER*^*T2*^*/Pkd1*^*fl/fl*^ mice were treated with tamoxifen or vehicle as previously described [9, 40]. Mice were culled at 16 weeks after tamoxifen treatment. The *Pax8rtTA; TetO-cre; Pkd1*^*fl/fl*^ were imported from the Baltimore PKD Centre [41] and rederived at the MRC Harwell. Deletion of the *Pkd1* gene was done as previously described, in brief, mice received intraperitoneal (IP) injections of doxycycline at postnatal days 10 and 11. *ANKHD1* knockout mice (*Ankhd1tm1a(KOMP)Wtsi*) were obtained from the Wellcome Trust Sanger institute (MGI:4847680), and rederived at the MRC Harwell. The *Ankhd1* knockout mice were generated as part of the International Mouse Phenotype Consortium [42] and were on a C57BL/6 background. We generated heterozygote double mutant *Ankhd1*^*+/−*^; *Pkd1*^*nl/nl*^ by intercrossing heterozygote *Pkd1*^*+/nl*^ male (C57BL/6) mice with heterozygote *Ankhd1*^*+/−*^ female mice. No *Pkd1*^*nl/nl*^; *Ankhd1*^*−/−*^ were obtained, suggesting that full *Ankhd1* loss in an ADPKD background is not viable.

### RNA immunoprecipitations

The Magna-RIP kit (17–700, Merck) was used to extract total protein and RNA following RNA immunoprecipitation (RIP) as per manufacturer’s instructions. Rabbit ANKHD1 antibody (HPA008718, Sigma) was used to pull down ANKHD1, and rabbit IgG (PP64B, Merck) was used as a negative control. The efficiency of pulldowns was confirmed by immunoblotting and only the samples where ANKHD1 was efficiently pulled down in the ANKHD1 RIP and not in the IgG RIP were included in further analysis. RNA was extracted from the immunoprecipitates using the Qiagen RNA extraction kit (RNeasy Mini, 74194, Qiagen).

### RNA-sequencing

RNA was extracted using the Qiagen RNA extraction kit (RNeasy Mini, 74194, Qiagen) from SKI001 and OX161c1 cells following either transfection with non-target RNAi (NTCsi) or ANKHD1 siRNA (ANKsi). The quality of RNA was assessed using Agilent’s Bioanalyser nanochip. High-quality RNA integrity number (RIN above 9) was used for total RNA sequencing, 10 million reads, 150 bp pair-end sequencing. The Fastq files underwent quality control, and the adapters and low-quality bases were trimmed. Reads were trimmed using TrimGalore (2) v 0.4.1. Cleaned Fastq files were mapped onto the reference human genome using STAR (3) v2.5.2a. Ensembl Homo_sapiens GRCh38 (release 95). BAM files were used to read counts in features which were calculated using HTSeq (6) v0.6.0. Any reads with mapping quality less than 10, or those that map to multiple loci or to overlapping gene regions were discarded to avoid ambiguity and false positive results. 27,377 genes were detected. Differential gene expression analysis was done using the counted reads and the R package edgeR (8) version 3.16.5 (R version 3.4.1) following pairwise comparisons.

### RNA extraction and qPCR

Total RNA was extracted using the miRNeasy Micro Kit (1071023, Qiagen) using the manufacturer’s protocol. RNA was treated with DNase I (AM2222, Invitrogen) for 20 min at room temperature to remove any contaminating DNA. DNase was then inactivated with 1 µL 25 mM EDTA pH8 heated for 10 min at 65 ^o^C. RNA was stored at -80 ^o^C. The isolated RNA was used to synthesise cDNA using the iScript kit (1708841, Biorad), which was then used in quantitative real-time PCR (qPCR) using SsoAdvanced Universal SYBR Green Supermix (1725274, Biorad). The primers used in qPCR to quantify mRNA expression were as follows: Cyclin D1 (*CCND1*), forward ATGAACTACCTGGACCGCTT, reverse TTCATTGAAATCGTGCGGGG; Cyclin Dependent Kinase 4 (*CDK4*), forward ATCAAGGATCTGATGCGCCA, reverse CACGGGTGTAAGTGCCATCT; Prostacyclin synthase (*PTGIS*), forward TTCCACATTACAGCCCCAGT, reverse CAGCACTGCATGGAGGTTG; β-actin, forward ATCATTGCTCCTCCTGAGCG, reverse GACAGCGAGGCCAGGATG.

### Mammalian cell culture & SiRNA transfections

SKI001 and OX161c1 are two conditionally-immortalised cell lines derived from the kidneys of patients with ADPKD [43, 44]. Cells were maintained in Dulbecco’s Modified Eagle Medium (DMEM) supplemented with 10% Foetal Bovine Serum (FBS) at 33^o^C. Cells were reverse transfected using Lipofectamine RNAi Max (Invitrogen) following manufacturer’s instructions, using 20nM final concentrations of single or pooled siRNAs (Horizon Discovery).

The pool Human ANKHD1 siRNA that was used included the following 4 siRNAs: si1 siGENOME SMARTpool siRNA D-014405-01- Target Sequence: GUAAAUUGCUAGAUGAAGG; si2 siGENOME SMARTpool siRNA D-014405-02 - Target Sequence: UGGCAGCUCUACUUAUUGA; si3 siGENOME SMARTpool siRNA D-014405-03 - Target Sequence: GCGCUAAUGUGCAUGCUAC; si4 siGENOME SMARTpool siRNA D-014405-04 - Target Sequence: ACACUGCGCUAACUUAUG, si2 and si4 were used individually as well. Cells were also reverse transfected with scrambled non-target control siRNAs (NTCsi), (20 nM non-targeting RNAi (D-001206-14.20, siGenome)). Comparisons were made between NTCsi versus ANKsi transfected cells that had undergone the same transfection protocol, using equal amounts of either ANKHD1 or non-target siRNA. The efficiency of ANKHD1 knockdown was measured by immunoblotting and/or qPCR and only transfection resulting in 70% or greater reduction in ANKHD1 were used for further analysis.

### Immunoblotting & antibodies

For immunoblotting whole cell lysates were generated by harvesting cells in lysis buffer in the presence of protease inhibitor cocktail (Roche, 04 693 116 001). All samples underwent a freeze-thaw cycle to ensure adequate cell lysis and were subjected to sonication (3 pulses of 10 s each). Lysed samples in 2X Laemmli buffer were boiled for 5 min, separated on 4–15% TGX SDS PAGE gels (Bio-Rad) and transferred to nitrocellulose membranes using the Trans-Blot Turbo Transfer System. The membranes were blocked in 5% skimmed milk in TBS for 30 min in a rotating platform at room temperature.

The antibodies that were used are: ANKHD1 Rabbit mAb (HPA008718, Sigma), p21 Waf1/Cip1 (12D1) Rabbit mAb (#2947, Cell Signaling), CDKN2D Rabbit polyclonal antibody (p19) (BS6940, Bioworld Technology), Cyclin D1 (E3P5S) XP^®^ Rabbit mAb (#55506, Cell Signaling), CDK4 (D9G3E) Rabbit mAb (#12790, Cell Signaling), phospho-RB, AQP1 Rabbit polyclonal antibody (Santa Cruz Biotechnology, sc-20810), AQP2 Goat polyclonal antibody (Santa Cruz Biotechnology, sc-9882), Ki67 Rabbit polyclonal antibody (ab15580, Abcam), β-actin Mouse mAb (ab8224, Abcam), goat anti-mouse IgG/HRP (P0447, Dako), goat anti-rabbit IgG/HRP (P0448, Dako).

### Cyst assays

3D in vitro cyst assays (generation of renal spheroids) were performed using the human ADPKD-derived cell lines SKI001 and OX161c1, which are tubular epithelial cell lines. Cells were reverse transfected with either 20 nM non-targeting RNAi (D-001206-14.20, siGenome) or 20 nM ANKHD1 RNAi (M-014405-00, siGenome) using Lipofectamine RNAimax (13778-075, Invitrogen) and Opti-MEM (31985-062, Gibco). After 24 h, RNAi-transfected cells were passaged and seeded into a 96 well plate 1:1 in Matrigel (E6909, Sigma) with 20 ng/mL EGF (Thermo Fisher Scientific, Cat No. 13247-051) and 200 ng/mL IFNα (human: pbl assay science, Cat No. 11100-1). The 96 well plate was incubated for 20 min at 33 °C to allow the Matrigel to polymerise, subsequently DMEM F12 medium with 10% FBS was added which was changed every 2 days. The remaining cells were plated onto a 12 well plate for immunoblotting. Cysts were imaged using the Leica Thunder Imager. Analysis of cyst size was performed using Image J. The scale measurement was set based on an automatically overlayed scale bar in the exported image from the Leica Thunder software and the diameter of the cyst was measured in µm using the straight-line measurement tool. Cysts that were out of focus were excluded from analysis.

### Serum urea nitrogen analysis

Blood samples were obtained in a 1.5 ml sterile tubes. Blood was left to coagulate at room temperature for 1 h. Serum was separated by centrifugation at 10,000 g for 10 min. Serum urea nitrogen analysis was performed at the Children’s Hospital, University of Sheffield.

### Immunostaining & microscopy

All tissue blocks were cut into 5 μm sections, deparaffinized, dehydrated, antigen-retrieved and stained overnight at 4^o^C using primary antibodies, followed by three quick washes and incubation with fluorescently labelled antibodies at room temperature for 6 h (Alexa fluor 488 goat anti-mouse IgG, #4408S, Cell Signalling, Alexa fluor 568 goat anti-rabbit IgG, A11036, Invitrogen). Imaging was performed using a Leica Thunder Imager.

### Statistical analysis

All experiments were repeated at least three independent times, unless otherwise stated. Data from independent experiments were imported into GraphPad Prism 9 and analysed either by unpaired, parametric Student’s *t* test for two-sample comparison or by ordinary one-way ANOVA for more than two samples, as indicated in relevant figure legends. Statistically significant values were considered at 0.05 or lower P value. Individual dots represent biological replicates.

## Results

### The PKD kidney is defined by excessive proliferation

**Fig. 1:**
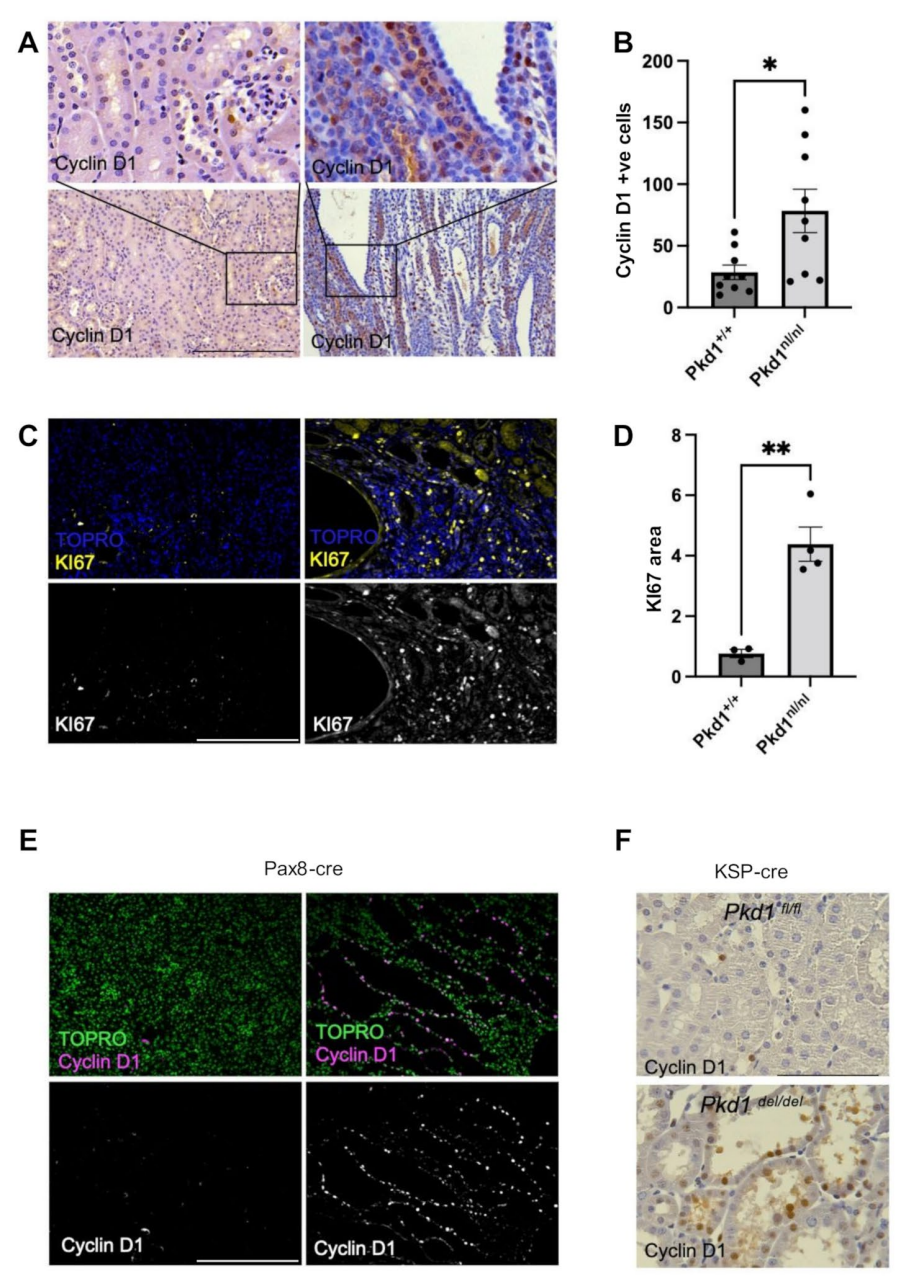
The ADPKD kidney is defined by increased proliferation. **A**) Cyclin D1 protein is increased in *Pkd1*^*nl/nl*^ mouse kidneys, determined by increased immunofluorescence staining using specific anti-Cyclin D1 antibodies (brown). Nuclear counterstain was achieved by haematoxylin staining (blue). *Pkd1*^*+/+*^ phenotype is shown on the left-hand side and *Pkd1*^*nl/nl*^ phenotype on the right-hand side. **B**) Quantification of Cyclin D1 was performed, and data are presented as ± S.E.M. **C**) Increased levels of Ki67 in *Pkd1*^*nl/nl*^ mice are shown using immunohistochemistry. TOPRO (blue) was used for nuclear counterstaining. *Pkd1*^*+/+*^ phenotype is shown on the left-hand side and *Pkd1*^*nl/nl*^ phenotype on the right-hand side. **D**) Quantification of Ki67 was performed, and data are presented as ± S.E.M. **E** and **F**) Tissue specific deletion of Pkd1 present increased levels of Cyclin D1 protein. **E**) Immunohistochemistry with Cyclin D1 shown in magenta. TOPRO (green) was used for nuclear counterstaining. *Pkd1*^*fl/fl*^ phenotype is shown on the left-hand side and *Pkd1*^*del/del*^ phenotype on the right-hand side. **F**) DAB immunostaning of kidney sections. Cyclin D1 is shown in brown. *Pkd1*^*fl/fl*^ phenotype is shown on the top panel and *Pkd1*^*del/del*^ phenotype on the bottom panel. Unpaired parametric *t*-test was used to calculate the indicated statistical significance, each dot represents an individual kidney. Scale bars are 500 μm

To characterise the extent of the proliferative defect, we examined the distribution and anatomical localisation of two well-established proliferation markers: Cyclin D1 and Ki67. Cyclin D1 is a marker of DNA synthesis, marking cells in the G1/S phase transition [45], while Ki67 is a protein expressed during all active phases of the cell cycle, except for quiescent cells (G0) that have exited the cell cycle [46]. These markers were chosen for their complementary roles in proliferation assessment and their widespread use in both research and clinical settings, offering the gold standard assessment of cellular proliferation. In the *Pkd1*^*nl/nl*^ mouse model of polycystic kidney disease, we observed a significant upregulation of proliferation markers in diseased kidneys. Immunohistochemical analysis of kidneys from multiple animals revealed a two-fold higher level of Cyclin D1 when compared to wild-type littermates (Fig. 1A-B). To corroborate these findings, we examined the expression of Ki67 levels, which were found to be threefold higher in *Pkd1*^*nl/nl*^ kidneys compared to wild-type littermates (Fig. 1C-D). The pronounced increase in both Cyclin D1 and Ki67 expression in the cystic epithelium provides robust evidence of enhanced proliferative activity, consistent with the hyperproliferative phenotype that is characteristic of human ADPKD.

To isolate any kidney-specific effects of *Pkd1* deletion in proliferation, we employed two conditional knockout mouse models where deletion of the *Pkd1* gene is under the control of either *Pax8-Cre* and *KSP-cre*. These models restrict *Pkd1* inactivation to renal tubular epithelial cells, eliminating potential confounding systemic effects, as *Pkd1* is expressed in multiple organs, such as the liver in addition to the kidney. Immunostaining for Cyclin D1 in both kidney-specific models showed a significant increase in proliferation (Fig. 1E-F) corroborating the findings obtained from the *Pkd1*^*nl/n*l^ model. The kidney-specific upregulation of proliferation in multiple models of murine ADPKD underscores the central role of proliferation in ADPKD pathogenesis. Moreover, it additionally suggests that intrarenal *Pkd1* loss is sufficient to drive the hyperproliferative phenotype. While potential paracrine effects cannot be excluded, these results highlight the primacy of kidney-intrinsic mechanism in ADPKD-associated proliferation.

### ANKHD1 is upregulated in ADPKD cystic epithelium

**Fig. 2:**
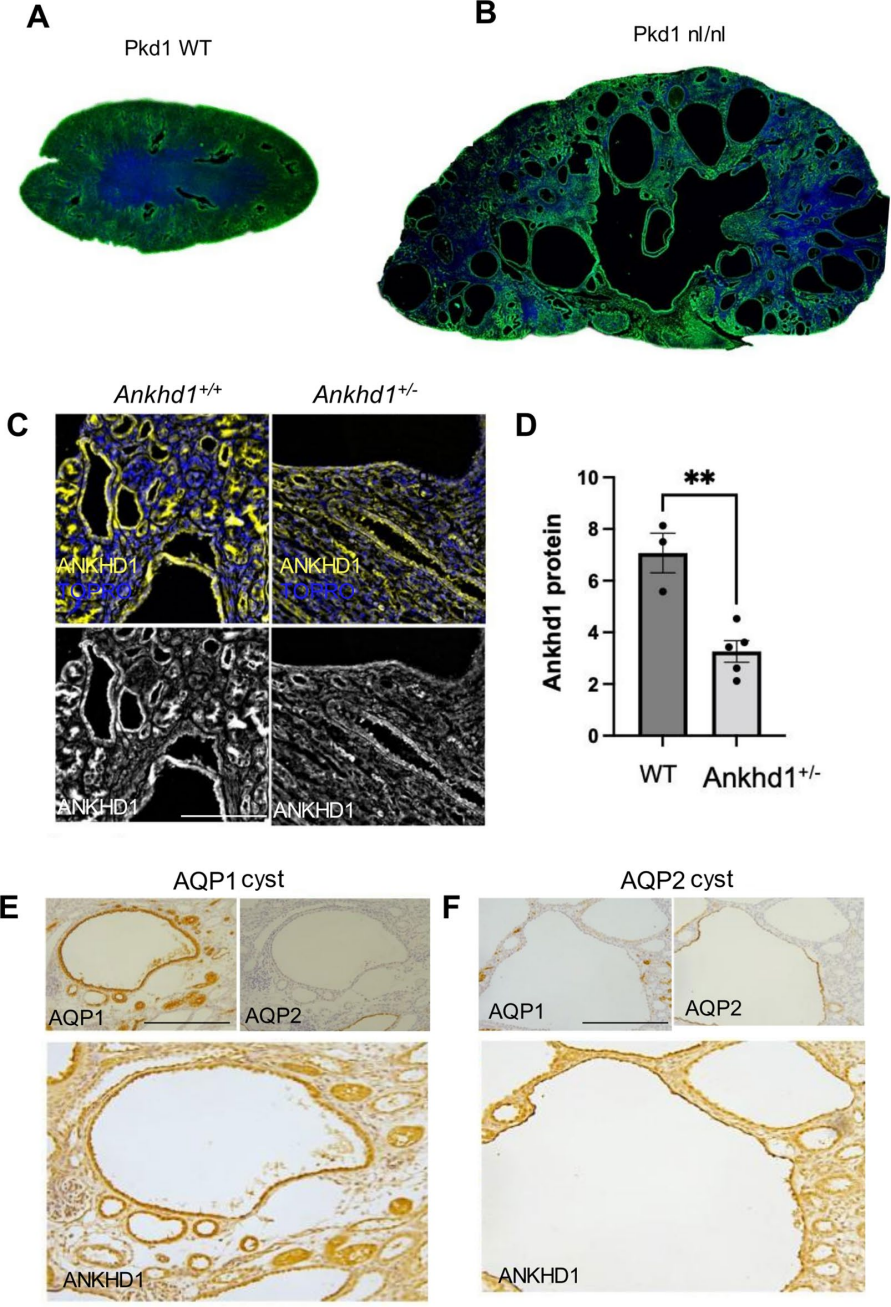
*Ankhd1*, a protein that controls proliferation, is expressed in epithelial cells of normal and ADPKD kidneys. Expression pattern of *Ankhd1* protein in (**A**) WT and (**B**) *Pkd1*^*nl/nl*^ mouse kidneys. ANKHD1 shown in green. TOPRO (blue) was used for nuclear counterstaining. **C**) ANKHD1 is highly expressed in cyst lining cells of *Pkd1*^*nl/nl*^*Ankhd1*^*+/+*^ mouse kidneys while is reduced in *Ankhd1*^*+/−*^ mice. **D**) Quantification of ANKHD1 was performed and data are presented as ± S.E.M. Each dot represents an individual mouse kidney. Student’s *t*-test was used to calculate the indicated statistical significance. ANKHD1 is expressed in proximal tubules of *Pkd1*^*nl/nl*^ mice as shown by Aquaporin-1 (AQP1) staining (**E**), and also in collecting ducts as shown by Aquaporin-2 (AQP2) staining (**F**). Scale bars are 500 μm

Building on our observation of elevated proliferation in ADPKD we decided to focus on ANKHD1. ANKHD1 is a critical regulator of cell proliferation in cancer, yet remains unexplored in ADPKD. ANKHD1’s potential significance in ADPKD is its known role as a driver of cancer and a significant regulator of pathways involved in ADPKD, such as JAK/STAT and YAP/TAZ. This makes ANKHD1 an attractive novel molecule to study in ADPKD. We investigated the expression of ANKHD1 in the *Pkd1*^*nl/nl*^ mouse. Global hypomorphic deletion of *Pkd1* was chosen as it closely recapitulates human ADPKD pathogenesis, including induction of fibrosis. Moreover, this particular model supports the hypothesis that lowering *Pkd1* expression below a threshold drives cytogenesis. Immunohistochemical analysis revealed widespread Ankhd1 throughout the kidney, with high levels in cyst lining cells of *Pkd1*^*nl/nl*^ mice (Fig. 2A-B). Ankhd1 was highly expressed in cystic epithelial cells, as well as the interstitium and the vasculature of the kidney. The specificity of the anti-ANKHD1 antibody was validated using kidneys from *Ankhd1*^*+/−*^ mice, which showed a 50% reduction in Ankhd1 protein levels (Fig. 2C-D). Further characterisation demonstrated Ankhd1 expression in both proximal and distal tubules, as confirmed by co-localisation with Aquaporin 1 (AQP1) and Aquaporin 2 (Aqp2) (Fig. 2E-F). The novel finding of Ankhd1 expression in ADPKD suggests its potential role in cystogenesis and disease progression, paralleling its known oncogenic functions in renal cell carcinoma and other cancers [37, 39, 47].

### ANKHD1 depletion attenuates cystogenesis in ADPKD

**Fig. 3:**
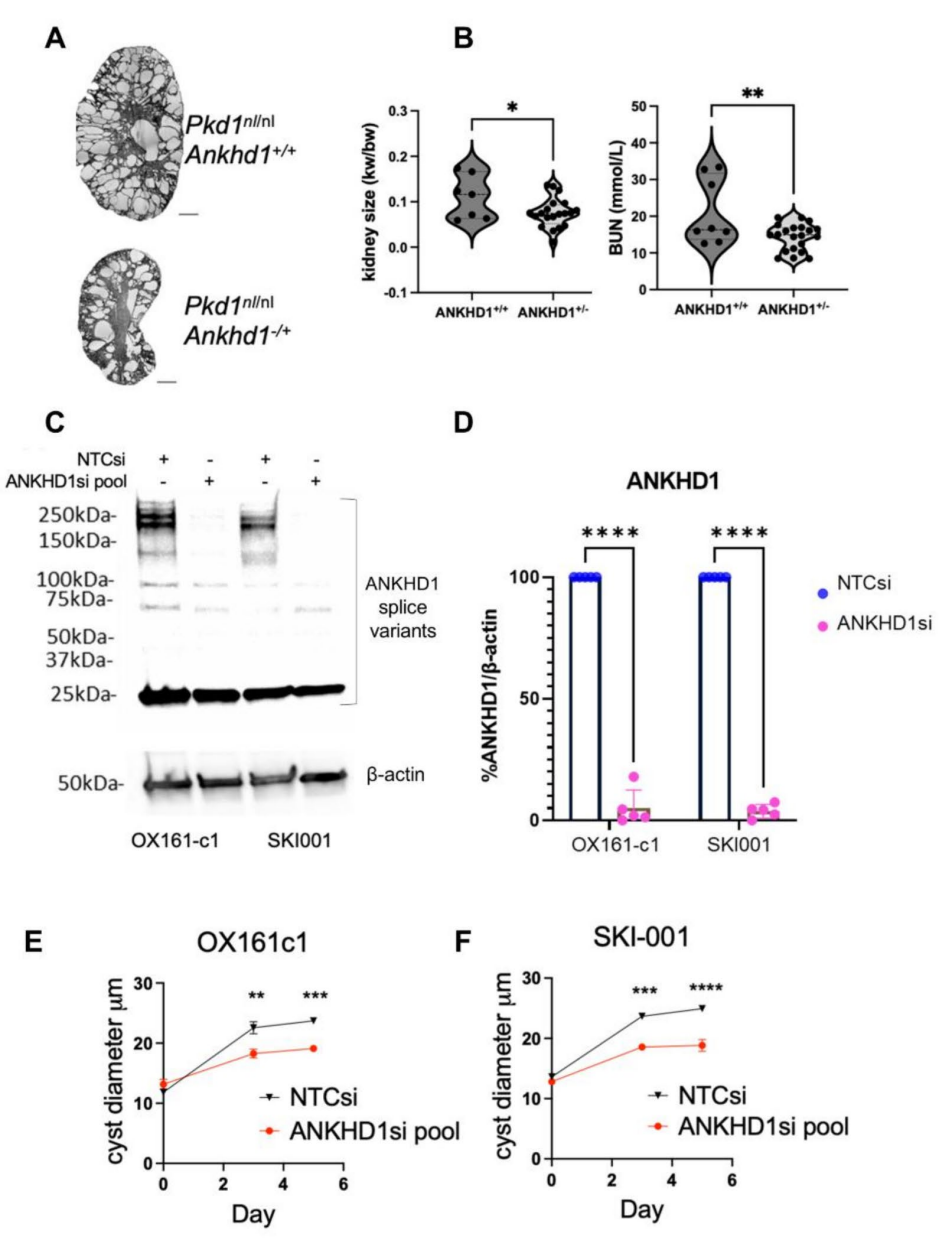
Loss of function of either human or murine ANKHD1 leads to reduced cyst size and improved renal function. **A**) H&E stained kidney sections from *Pkd1*^*nl/nl*^ mice with either two *Ankhd1* copies (*Ankhd1*^*+/+*^) or with hemizygous deletion of *Ankhd1* (*Ankhd1*^*+/−*^). Scale bars are 1,000 μm. **B**) The graphs show the kidney weight over body weight ratio and blood urea nitrogen for *Ankhd1*^*+/+*^ and *Ankhd1*-deficient mice analysed at 4 weeks of age. Each dot represents an individual mouse kidney. Data are shown as ± S.E.M. *t*-test statistical analysis was performed with p values *<0.05 and **<0.001. **C**) Successful knock-down of human ANKHD1 protein in two patient derived cell lines. The predicted band size of ANKHD1 is 270 kDa, with some laddering expected. There is a non-specific band at 25 kDa. **D**) Western blot quantification of human ANKHD1 knock-down. Dots represent independent biological replicates. Cyst diameter (µm) was measured in **E**) OX161c1 and **F**) SKI001 cells after knocking-down ANKHD1 (ANKHD1si pool) or the use of a non-target-control siRNA (NTCsi). P values: *<0.05, **<0.01, ***<0.001 and ****<0.0001. Dots represent independent biological replicates

To investigate the functional role of ANKHD1 in ADPKD progression, we employed both in vivo and in vitro approaches. In vivo, we generated *Pkd1*^*nl/nl*^ mice heterozygous for *Ankhd1*, which exhibited reduced kidney size, fewer cysts and improved renal function as evidenced by lower blood urea nitrogen (BUN) (Fig. 3A-B). By comparison, BUN in wild-type littermates was 6.54 mmol/L with SD of 0.3790 (*n* = 6 mice), hence *Ankhd1*^*+/−*^ mice showed a improved kidney function but did not reach wild-type levels. This suggests that *Ankhd1* reduction ameliorates the ADPKD phenotype by partially restoring kidney function, without fully reversing disease..

For in vitro studies, we utilised the patient-derived human renal tubular epithelial cell lines SKI001 and OX161c1 [43]. *ANKHD1* was efficiently silenced using a pool of 4 specific siRNAs, achieving 70% reduction in protein levels (Fig. 3C-D), using a protocol previously established in the laboratory [40]. To assess the impact of *ANKHD1* knockdown on cystogenesis, we performed 3D cyst formation assays, which mimic the in vivo cystic microenvironment [48]. *ANKHD1*-silenced cells formed significantly smaller cysts in both cell lines, as measured by cyst diameter, indicating overall reduced spheroid size (Fig. 3E-F). These complementary in vitro and in vivo findings demonstrate for the first time that ANKHD1 is a key driver of cystogenesis in ADPKD. Its reduction leads to attenuated cyst formation and improved renal parameters, highlighting ANKHD1 as a potential therapeutic target for ADPKD.

### Unbiased transcriptome analysis reveals ANKHD1 as a key regulator of proliferation in ADPKD

**Fig. 4:**
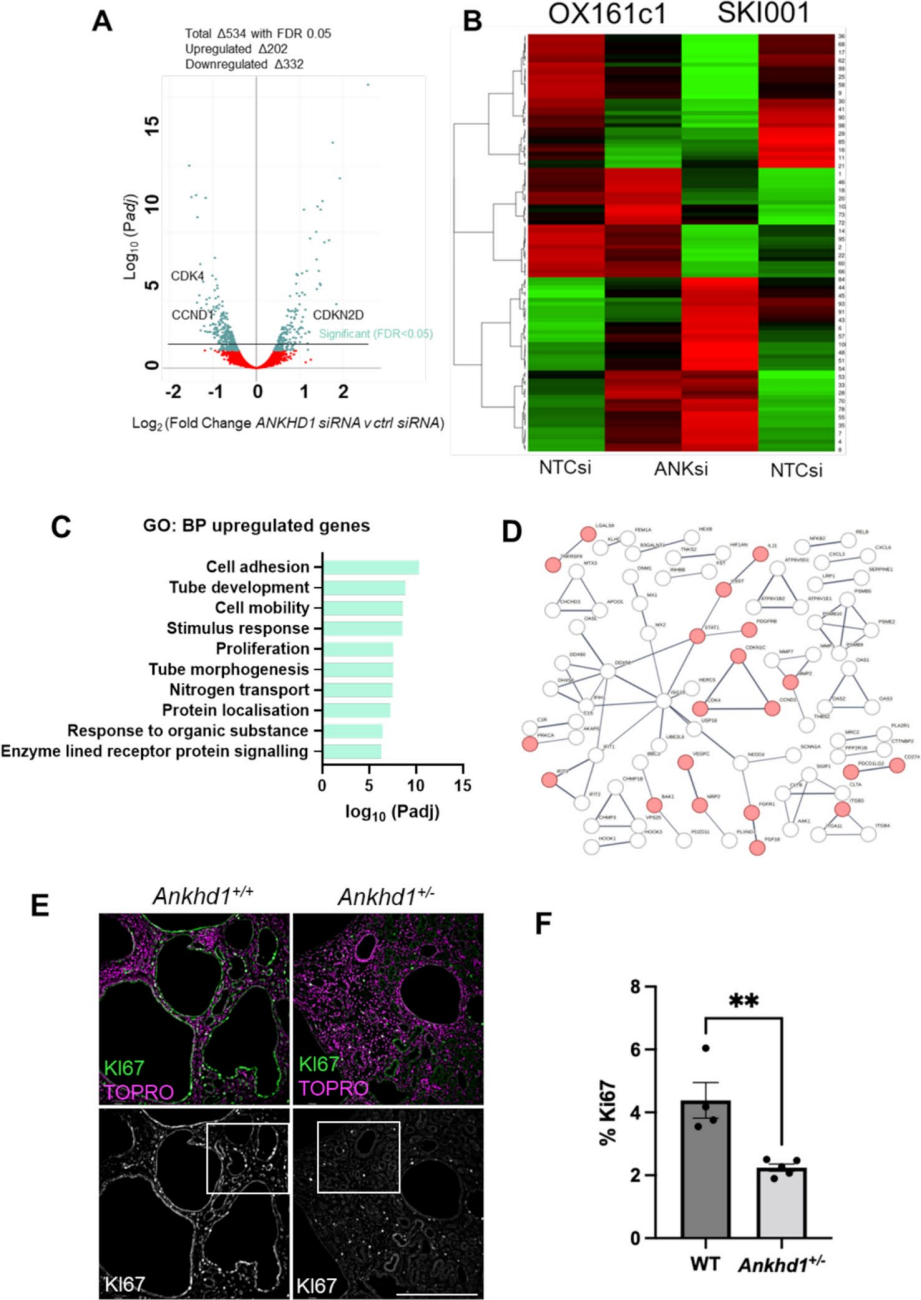
RNA sequencing analysis reveals a major role for ANKHD1 in the control of proliferation. **A**) ANKHD1-siRNA whole transcriptome profiling in two renal epithelial cell lines (OX161c1 and SKI001) was performed using next-generation NextSeq500 Illumina platform. Volcano plots of genes with differential expression in Ankhd1-high (non-targeting siRNA) versus Ankhd1-low cells (Ankhd1-siRNA) were plotted (adjusted *P* ≤ 0.01 and fold change ≥ 0.5). P values were calculated with two-sided likelihood ratio test and adjusted by Benjamini-Hochberg method; *n* = 4 (2 conditions Ankhd1-siRNA and nontarget siRNA and 2 biological samples). **B**) Heatmap shows 534 significantly (FDR ≤ 0.05) differentially expressed genes for the two human cell lines. Each row represents the z-score values of one differentially expressed gene across the samples (green, low expression; red, high expression). **C**) GSEA analysis showing the top 10 most enriched pathways that are positively regulated by *ANKHD1.***D**) STRING analysis showing interaction networks differentially expressed proteins involved in proliferation highlighted in red. **E**) Immunostaining of *Pkd1*^*nl/nl*^*Ankhd1*^*+/+*^ (left hand panel) and *Pkd1*^*nl/nl*^*Ankhd1*^*+/−*^ (right hand panel) mouse kidneys reveals enhanced proliferation in mice with wild-type expression of ANKHD1 when compared to ANKHD1-deficiency (*Ankhd1*^*+/−*^). Ki67, shown in green, was used as a proliferation marker. TOPRO (magenta) was used for nuclear staining. Scale bar denotes 500 μm. **F**) Quantification of % of Ki67 positive cells is presented as ± S.E.M. Each dot represents an individual mouse kidney. Unpaired parametric *t*-test was used to calculate the indicated p-value

To gain mechanistic insight into how ANKHD1 promotes cystogenesis, we took an unbiased approach using next-generation RNA sequencing. We analysed ADPKD-derived epithelial cells with and without ANKHD1 silencing achieving a 70% or greater reduction of ANKHD1 levels. High quality of RNA (RIN > 9) was sequenced, revealing 534 differentially expressed genes upon ANKHD1 knockdown (Fig. 4A-B) and (sup T1).

Gene set enrichment analysis (GSEA) of ANKHD1-regulated genes highlighted ‘proliferation’ as a key pathway, along with ‘cell adhesion’, ‘tube formation’ and ‘cell mobility’– all implicated in ADPKD pathogenesis (Fig. 4C). Notably, we observed downregulation of CDK4 and upregulation of p19 (CDKN2D) upon ANKHD1 silencing. String analysis of significantly altered genes placed proliferation regulators (CDK4, CCND1 and CDKN1C) at the centre of protein-protein interaction network. Cell transfection with siRNAs did not cause cytotoxicity or cellular death (Sup Fig. 1A-B).

To validate these findings in vivo, we performed immunohistochemistry on *Pkd1* null mice with wild-type *Ankhd1* (*Ankhd1*^*+/+*^) or with heterozygote deletion of *Ankhd1 *(*Ankhd1*^*+/−*^). Ki67 staining revealed a greater than 50% reduction in proliferating cells in *Ankhd1*^*+/−*^ mice (Fig. 4E-F) confirming ANKHD1’s crucial role in controlling ADPKD cell proliferation. These results collectively indicate that ANKHD1 is a key regulator of proliferation in ADPKD, providing new insights into the mechanisms of cystogenesis.

### ANKHD1 regulates CDK4 expression in ADPKD models via direct mRNA interaction

This section builds on our previous transcriptomics findings, focusing on the mechanistic relationship between ANKHD1 and CDK4 in ADPKD. The RNA-seq revealed that CDK4 is under the control of ANKHD1, whereby lowering ANKHD1 reduces the mRNA levels of CDK4, prompting further investigation into this critical regulator of cell cycle.

**Fig. 5:**
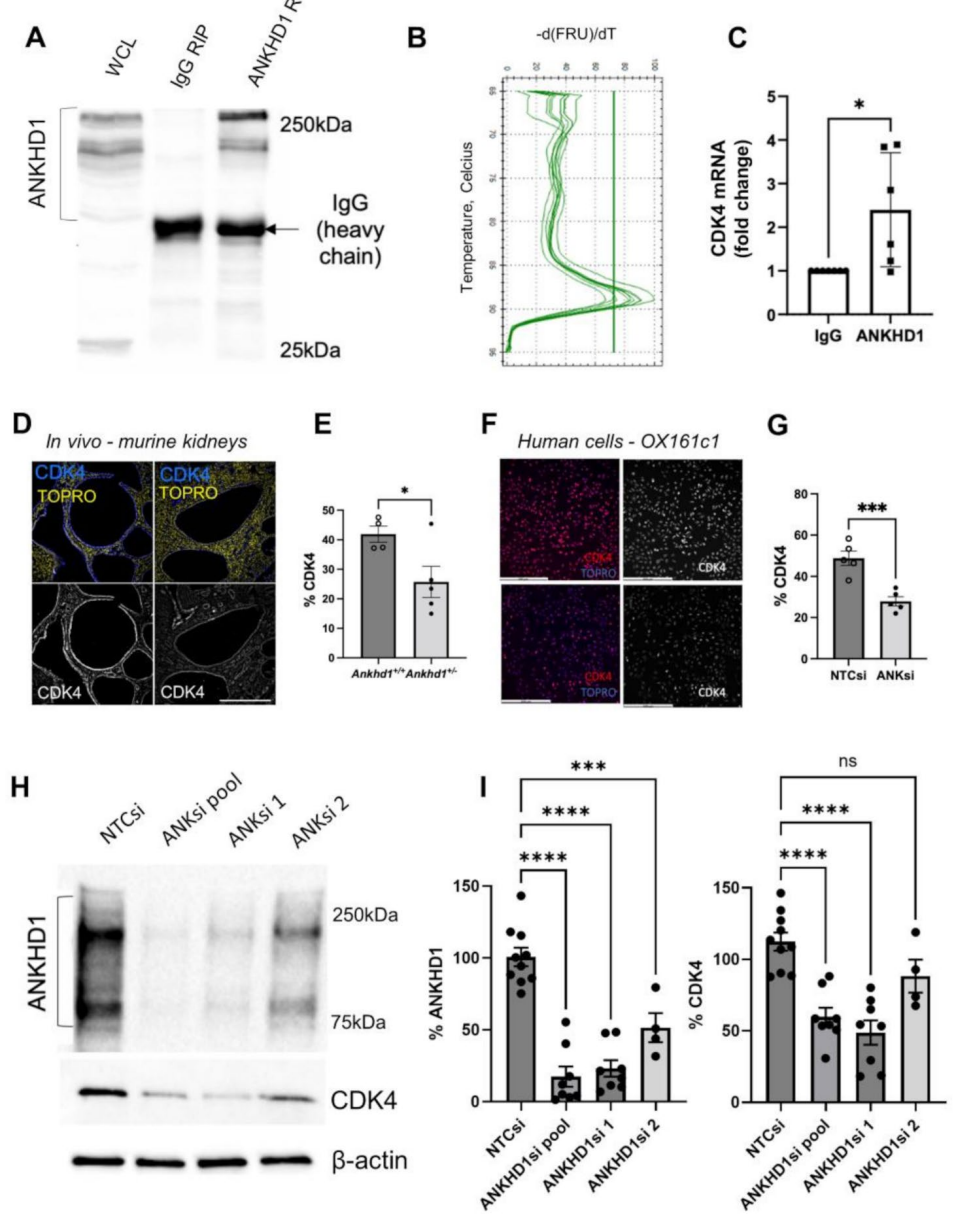
ANKHD1 binds to CDK4 mRNA and its loss leads to reduced CDK4 expression in vitro and in vivo. **A**) ANKHD1-probed immunoblots of RNA immunoprecipitations (RIPs). Left hand-side lane is the 10% input (without RIP), middle lane is the RIP with a control rabbit IgG antibody (not pulling down ANKHD but showing the IgG heavy chain band) and on the right-hand lane is the ANKHD1-RIP showing both the specific ANKHD1 bands and also the non-specific IgG heavy chain. **B**) RNA associated with either IgG or ANKHD1 pull down was extracted and used to generate a melting curve plot for CDK4 mRNA by qPCR. **C**) qPCR showing CDK4 mRNA from either IgG-RIP or ANKHD1-RIP. Dots represent independent biological replicates. **D**) CDK4 protein is reduced in *Pkd1*^*nl/nl*^ mice with the hemizygous deletion of *Ankhd1* (*Ankhd1*^*+/−*^) as shown by immunohistochemistry. CDK4 shown in blue. TOPRO (yellow) was used for nuclear staining. Scale bars are 500 μm. **E**) Quantification of % of CDK4 positive cells is presented as ± S.E.M., Student’s *t*-test was used to calculate the indicated p-value. Dots represent individual mouse kidneys. **F**) Immunocytochemistry showing reduced CDK4 expression in the patient-derived OX161c1 cells where *ANKHD1* was knocked-down. Scale bars are 500 μm. The corresponding quantification of CDK4 is shown (**G**), dots represent independent biological experiments. **H**) Western blot analysis showing full-length ANKHD1 using a non-target siRNA (lane 1), a pool of four siRNAs (lane 2) and two individual siRNAs (lane 3 and 4). Below, the corresponding CDK4 levels are shown. β-actin was used as loading control. **I**) Quantification of % knock-down of ANKHD1 (left) and CDK4 (right) levels, presented as ± S.E.M. One-way ANOVA was used to calculate statistical significance. Dots represent independent biological experiments

We utilised both in vitro and in vivo models to explore the ANKHD1-CDK4 relationship. Immunostaining of *Pkd1*^*nl/nl*^ mice heterozygous for Ankhd1 showed reduced Cdk4 expression compared to *Ankhd1*^*+/+*^ littermate controls (Fig. 5D-E), corroborating the RNA-seq results. This finding was further substantiated in vitro using human patient-derived cells (OX161c1), where knockdown of ANKHD1 resulted in greater than 40% reduction in CDK4 protein levels (Fig. 5F-G). Immunoblotting using either a pool of four siRNAs or two individual siRNAs targeting ANKHD1 confirmed the downregulation of CDK4 upon ANKHD1 silencing (Fig. 5H-I). The different effects observed with individual siRNAs highlight the importance of using multiple silencing siRNAs to ensure robust results. Specifically, while most siRNA treatment resulted in CDK4 reduction, ANKHD1si 2 did not have the same effect, possibly because it did not effectively reduce the levels of ANKHD1 (Fig. 5H-I).

To elucidate the mechanism underlying ANKHD1’s control of CDK4 mRNA expression, we performed RNA immunoprecipitation (RIP) analysis. This technique revealed a significant enrichment of CDK4 mRNA in ANKHD1 immunoprecipitates compared to irrelevant IgG controls (Fig. 5A-C), suggesting an interaction between ANKHD1 protein and CDK4 mRNA. 

These findings collectively demonstrate that ANKHD1 positively regulates CDK4 mRNA levels both in vitro and in vivo, likely through direct protein-mRNA interactions. This interaction is presumably mediated by ANKHD1’s KH (RNA binding) domain, although further studies are needed to confirm this hypothesis. Taken together, our results provide novel insights into the molecular mechanisms underlying ADPKD progression, highlighting the ANKHD1-CDK4 axis as a potential therapeutic target. This works aligns with previous work showing ANKHD1’s role in regulating proliferation in cancer, where ANKHD1 was found to control cell cycle progression via miRNA interactions requiring its KH domain [47].

### ANKHD1 regulates the Cyclin D1/CDK4/pRb axis in ADPKD via p19 modulation

**Fig. 6:**
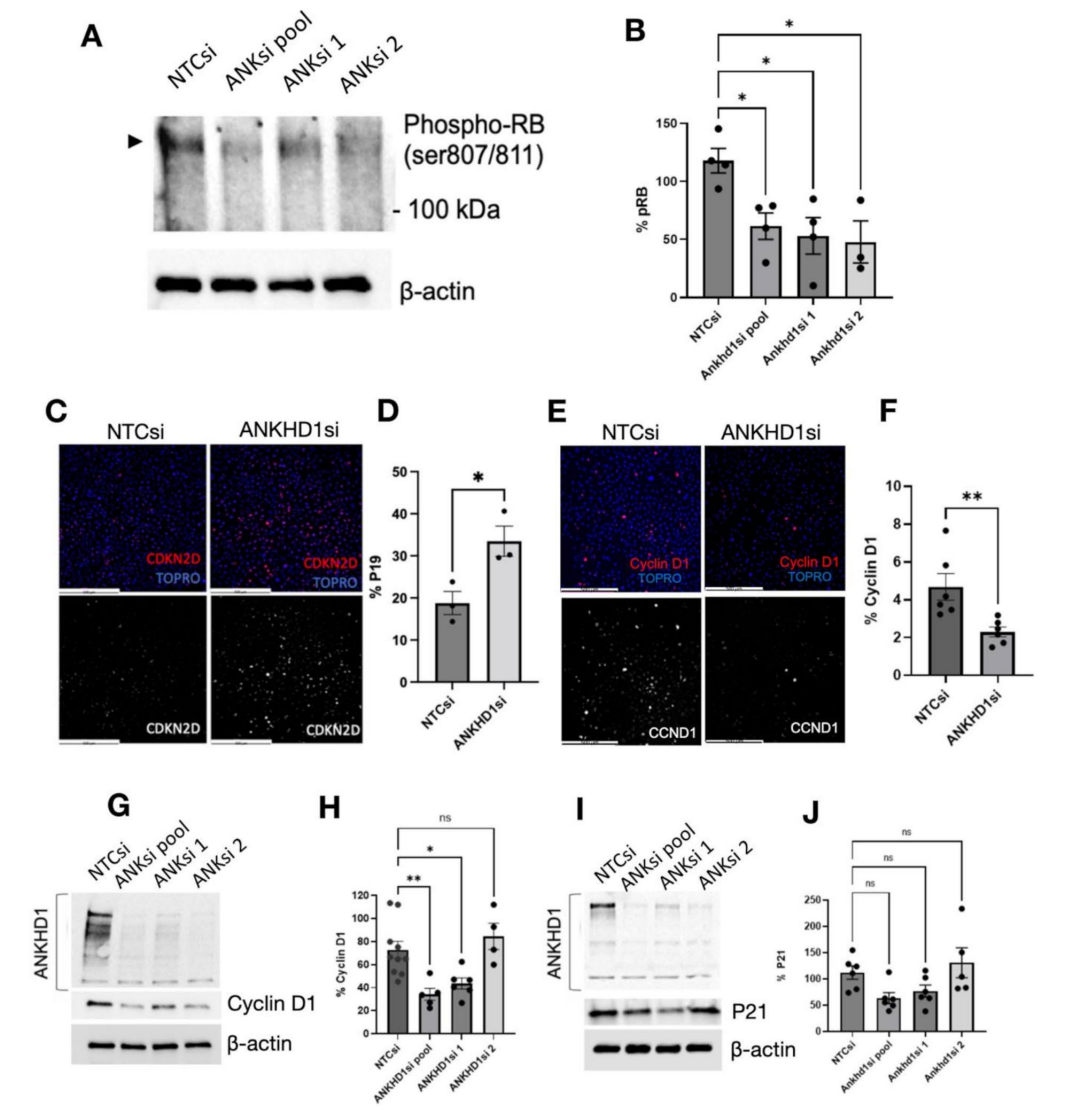
ANKHD1 loss leads to reduced proliferation via a CDK4, p19, pRb dependent, but p21 independent pathway. **A**) Western blot analysis showing phosphorylated Retinoblastoma (pRb, indicated by the arrowhead) protein levels after silencing with a non-target siRNA (lane 1), a pool of four siRNAs (lane 2) and two individual siRNAs (lane 3 and 4). β-actin (lower panel) was used as loading control. **B**) Quantification of % pRb levels. Dots represent independent biological replicates, presented as ± S.E.M. One-way ANOVA was used to calculate statistical significance. Dots represent biological replicates. **C**) Immunocytochemistry showing upregulation of CDKN2D (p19) protein expression in the patient-derived OX161c1 cells where *ANKHD1* was knocked down. Scale bars are 500 μm. (**D**) p19 quantification, dots represent biological replicates. **E**) Immunocytochemistry showing downregulations of CCND1 (Cyclin D1) protein expression in the patient-derived OX161c1 cells where *ANKHD1* was knocked down. Scale bars are 500 μm. **F**) Cyclin D1 quantification, dots represent biological replicates. **G**) Western blot analysis showing full-length ANKHD1 using a non-target siRNA (lane 1), a pool of four siRNAs (lane 2) and two individual siRNAs (lane 3 and 4). Below, the corresponding Cyclin D1 levels are shown. β-actin was used as loading control. **H**) Quantification of % Cyclin D1 levels normalised against β-actin, presented as ± S.E.M, each dot represents an independent biological experiment. One-way ANOVA was used to calculate statistical significance. **I**) Western blot analysis showing full-length ANKHD1 using a non-target siRNA (lane 1), a pool of four siRNAs (lane 2) and two individual siRNAs (lane 3 and 4). Below, the corresponding p21 levels are shown. β-actin was used as loading control. **J**) Quantification of % p21 levels normalized against β-actin, presented as ± S.E.M, each dot represents an independent biological experiment. One-way ANOVA was used to calculate statistical significance

Given that the Cyclin D1/CDK4 protein complex is responsible for the hyper-phosphorylation of the retinoblastoma protein (p-Rb) [49], we examined the phosphorylation levels of pRb. We found that ANKHD1 silencing led to a significant reduction in retinoblastoma (Rb) phosphorylation, a critical step in cell cycle progression. The decreased levels of pRb are likely mediated by decreased availability of the Cyclin D1/CDK4 complex, as evidenced by reduced phospho-Rb levels (Fig. 6A-B).

Importantly, we observed a significant increase in p19 expression, an INK4 family member and CDK4 inhibitor, upon ANKHD1 depletion (Fig. 6C-D). Moreover, as expected we observed a significant decrease in the levels of Cyclin D1 when ANKHD1 was silenced, observed both by immunocytochemistry (Fig. 6E-F) and confirmed by immunoblotting (Fig. 6G-H). These data together suggest that ANKHD1 negatively regulates the cell cycle inhibitor p19, while it positively regulates Cyclin D1/CDK4, providing a novel mechanism for its pro-proliferative effects. Interestingly, p21, another cyclin-dependent kinase inhibitor, remained unaffected, indicating specificity in ANKHD1’s regulatory role (Fig. 6I-J).

These findings are clinically relevant as they identify ANKHD1 as a potential therapeutic target in ADPKD. By modulating the Cyclin D1/CDK4/Rb axis and p19 regulation, ANKHD1 inhibition could potentially slow cyst growth and disease progression. This work provides further insights into the molecular mechanisms underlying ADPKD pathogenesis, identifying ANKHD1 as a key contributor.

## Discussion

This study reveals ANKHD1 as a novel regulator of proliferation in ADPKD, offering new insights into disease progression and potential therapeutic strategies. We demonstrate that *Pkd1* loss leads to cell cycle defects and elevated proliferation in three independent mouse models of ADPKD. Importantly, we identify ANKHD1 as a key mediator of this aberrant proliferation controlling the Cyclin D1/CDK4 axis through binding to CDK4 mRNA. While previous studies have identified proliferative pathways such as mTOR and JAK/STAT as key regulators of proliferation in ADPKD, our study is the first to identify ANKHD1 as a direct regulator of the Cyclin D1/ CDK4/ p19 pathway, revealing a novel mechanism of cystic epithelial cell proliferation. Moreover, unlike previously characterised secondary messengers, such as cAMP, we show that ANKHD1 exerts its effects via direct physical binding to *CDK4* mRNA. This is a previously unrecognised mechanisms linking RNA-binding to ADPKD pathogenesis. Taken together, this study provides the first evidence that ANKHD1 targeting not only reduces proliferation but also improves renal function in vivo. These findings suggest that ANKHD1 is not just a marker of disease progression but a potential therapeutic target.

These findings expand our understanding of ADPKD pathogenesis beyond the currently available treatment, tolvaptan [50]. While tolvaptan slows disease progression and has proven that ADPKD is indeed a disease that can be modified, it is poorly tolerated due to common side-effects (polyuria) and requires regular monitoring due to idiosyncratic adverse effects i.e. hepatotoxicity [51]. Our study suggests that targeting ANKHD1, or its downstream effectors, could provide an alternative therapeutic avenue. While ANKHD1 is an RBP and RBPs do not belong to the ‘druggable genome’, recent advances in the use of siRNA as medicines (reviewed previously [52]) open the opportunity to expand therapies to previously unexplored targets, such as ANKHD1 and its mediators.

Interestingly, proliferating cells were found both in the growing epithelial derived cysts, in the non-cystic epithelium but were also detected in the interstitium. The presence of proliferation in the interstitium suggests that there there is contribution to disease from these layers. If proliferation was to be blocked, its therapeutic potential may be via affecting both the epithelial and non-epithelial layers of the kidney. While tissue-specific deletion is a genetically accurate tool (KSP and Pax8 drivers), ADPKD in humans is a disease that affects multiple organs, not only the kidneys, and therefore analysis of results from kidney-specific models should be performed with caution and in combination with hypomorphic mutants such as the *Pkd1*^*nl/nl*^ used in this study. Hence a future ideal therapeutic strategy should include targeting of multiple cells and not only certain segments of the tubules (such as AQP1/2 positive cells).

Since the development of cysts in ADPKD is at least in part due to increased cell proliferation [53] and ANKHD1 is a cancer associated pro-proliferative protein [54, 55] we hypothesised that ANKHD1 has a major role in controlling proliferation in the polycystic kidney. Indeed, our NGS and RIP experiments combined with immunostaining for Ki67, confirmed this hypothesis. Using RNA immunoprecipitation assays, we uncovered that ANKHD1 controls proliferation by binding to CDK4 mRNA. Moreover, lowering ANKHD1 leads to reduced CDK4 and Cyclin D1 levels. However, it should be noted that cystogenesis is a multifaceted process that extends beyond altered cellular proliferation. While increased proliferation of cyst-lining epithelial cells is a hallmark feature, other mechanisms such as altered cell migration, defects in cytoskeletal organization, and disrupted signalling pathways also play critical roles. For instance, abnormalities in actin cytoskeleton dynamics, regulated by proteins like PC1, contribute to defective cell migration and structural changes in renal tubules. Taken together, in ADPKD patient-derived human renal epithelial cells, ANKHD1 promotes CDK4 /Cyclin D1 /p19-dependent proliferation, yet whether its role extends to other functions (such as polarity and migration) is currently unknown.

To confirm whether ANKHD1 is indeed a key regulator of the CDK4 pathway also in vivo, we examined this regulation in mice. We found that ANKHD1 binds to CDK4 mRNA and its loss leads to reduced CDK4 expression in vivo, in a level similar to that observed in human epithelial cells. This suggests that the role of ANKHD1 is to control the levels of CDK4 and hence overall cell cycle progression in renal epithelial cells in vitro and in vivo, making ANKHD1 a novel regulator of renal cell proliferation. This is intresting as transient inhibtion of CDK4 has been shown to protect renal epithelial cells from DNA damage and apoptosis during acute kidney injury [56]. Moreover, persistent CDK4 activation has been shown to lead to chronic tubular injury and fibrosis establishment [57]. Therefore, while here we explore the role of ANKHD1 in ADPKD, we propose that may have additional roles in diseases beyond ADPKD, such as in both acute and chronic kidney diseases.

While it was already known that ANKHD1 is highly expressed in kidneys of patients with renal cell carcinoma [35], whether it was expressed in normal kidneys without disease and in polycystic kidneys was previously unknown. Here we show that ANKHD1 is indeed expressed in non-cancer kidney tissue, and its expression is high in the polycystic kidney. Cysts can arise from both proximal and distal portions of the tubule in the kidney [58]. We found strong ANKHD1 expression in both segments, suggesting that any effect of ANKHD1 on proliferation will affect both proximal and collecting ducts equally. This is important becasue it offers comprehensive disease management, as ANKHD1 is indeed present and functional in both segments of the kidney where cysts arise. By targeting ANKHD1 we can potentially target simultaneously multiple sites of ADPKD pathology. Moreover, the fact that ANKHD1 is also expressed in proximal tubules is of further importance, as proximal epithelial cells often fail to repair and hence contribute to fibrosis in multiple renal pathologies.

Elevated activity from the Cyclin D1 /CDK4 complex axis induces retinoblastoma protein (pRb) hyperphosphorylation leading to its inactivation and triggering uncontrolled cell proliferation [59, 60]. Accordingly, the downregulation of CDK inhibitors (such as p19 and p21) is a common occurrence in human tumours [61]. Here, we show that ANKHD1 controls the level of cell cycle progression by stabilising the levels of CDK4/CyclinD1 thus promoting proliferation, while it increases levels of the cell cycle inhibitor, p19, resulting in reduced proliferation rates. By knocking down ANKHD1, we achieve lower levels of Cyclin D1/CDK4 complex thus reducing the CDK4-mediated phosphorylation of Rb protein. Unphosphorylated Rb remains bound to E2F in an inactive complex, which keeps cell cycle in the G1 phase, stopping the cell from entering division, thereby reducing proliferation of cystic cells. Likewise, a reduction in ANKHD1 levels results in an increase in the cell cycle inhibitor p19, which binds to the Cyclin D1/CDK4 complex and consequently blocks phosphorylation of Rb. This dual mechanism leads to cell cycle arrest in G0/G1 phase and a lower level of proliferation. Collectively, these results underscore the critical role of ANKHD1 in driving cell cycle dynamics in ADPKD and highlight its potential as a therapeutic target in modulating cyst growth.

Human renal epithelial cells have a finite proliferative potential, and they eventually lose their ability to proliferate and restrict their growth, entering a state known as cellular senescence or quiescence, depending on the context [62]. Yet, renal epithelial cells, with *Pkd1* mutations, remain hyperproliferative for longer than expected. The mechanisms that allow *Pkd1* mutant cells to avoid senescence in the absence of oncogenic transformation are currently unknown. Here we show that ANKHD1 enhances Cyclin D1/CDK4 and at the same time reduces p19, thus prolongs the time that cells are undergoing active proliferation. We suggest that by reducing the expression levels of ANKHD1, we could achieve slower proliferation, reduce the size of the renal cysts and help slow down kidney function decline in patients with ADPKD. The effect of reduced ANKHD1 expression over time requires to be investigated to ensure cells retain their ability to repair.

While our study provides significant insights into the role of ANKHD1 in ADPKD, several limitations need to be considered. Firstly, our findings are primarily based on murine models and human cell lines. While these are disease relevant, they do not fully recapitulate the complexity of human disease. Future studies using human tissues and/or additional in vivo models could enhance the generalisation of the results. Additionally, our in vivo results are confounded by the genetic background of the mouse models used, particularly the *Pkd1*^*nl/nl*^ mice (C57BL/6), which carry genetic variations that could affect disease progression. Moreover, environmental factors such as diet and housing conditions can also impact kidney function and cyst development obscuring any specific effects of ANKHD1. Lastly, while we identified and characterised a critical role between ANKHD1 and CDK4/p19 mRNAs, other potential pathways and interactions may also contribute to ADPKD progression. We note that the patient-derived cell lines used here were p53 inactive, hence any potential role of p53 could not be studied. Despite this limitation, ANKHD1 silencing rescued proliferation in patient-derived cells even in this p53 null genetic background, suggesting that p53 is not necessary for its actions. While this study establishes ANKHD1 as a key regulator of proliferation in ADPKD, further research is needed to validate its role in human patient samples, explore its interactions with other cystogenic pathways, and assess feasibility of targeting ANKHD1 therapeutically using siRNA based therapies and/or going downstream of ANKHD1. Moreover, studies to identify efficient delivery of RNA therapeutics specifically into kidneys are critical.

## Conclusions

In conclusion, our study discovers ANKHD1 as a novel regulator of proliferation in ADPKD, bridging the gap between aberrant cellular proliferation and disease progression. We show that loss of function of either human or murine ANKHD1 leads to smaller kidneys, reduced cyst size and improved renal function. Mechanistically, we reveal that ANKHD1 controls the Cyclin D1/CDK4 / p19 pathway. Mechanistically ANKHD1 silencing employs two mechanisms to control proliferation (i) reduces the mRNA of CKD4 / Cyclin D1 and (ii) increases the mRNA of p19. Collectively these two mechanisms lead to altered phosphorylation of Rb and reduced cell cycle progression.. As ADPKD is an incurable condition with only one therapeutic treatment, our findings open new avenues for therapeutic intervention. Future research should focus on developing specific and safe modulators of ANKHD1 and evaluate their efficacy in preclinical models.

## Data Availability

All data including supporting datasets are made available as main figures or supplementary information files.

## Abbreviations

ADPKD: Autosomal Dominant Polycystic Kidney Disease
ANKHD1: Ankyrin Repeat and single KH Domain 1
AQP1: Aquaporin-1
AQP2: Aquaporin-2
CKD: Chronic Kidney Disease
pRb: Phosphorylated Retinoblastoma
RBPs: RNA- binding proteins
RIP: RNA immunoprecipitation
